# Effects of cluster nursing on cardiac function and quality of life in coronary heart disease patients with chronic heart failure: A protocol of randomized controlled trial

**DOI:** 10.1097/MD.0000000000029091

**Published:** 2022-04-08

**Authors:** Qian Jin, Yi Zhou, Delu Yin, Hong He, Yonghua Liu, Yiling Wu

**Affiliations:** The First People's Hospital of Lianyungang, Department of Cardiovascular Medicine, No. 182 North Tongguan Road, Lianyungang, Jiangsu Province, P.R. China.

**Keywords:** chronic cardiac failure, cluster nursing, coronary heart disease, protocol, randomized controlled trial

## Abstract

**Background:**

Coronary heart disease (CHD) chronic heart failure has high morbidity and mortality, which poses a serious threat to patients’ quality of life and life safety. For the treatment of chronic heart failure of CHD, in addition to drugs, high quality nursing measures are also very important. Cluster nursing is a high-quality nursing model based on evidence-based evidence. There is no clinical study to evaluate the effect of cluster nursing on cardiac function and quality of life of CHD patients with chronic heart failure.

**Methods:**

This is a prospective randomized controlled trial to investigate the effects of cluster nursing on cardiac function and quality of life in patients with CHD chronic heart failure. Approved by the Clinical Research Ethics Committee of our hospital, patients will be randomly assigned to either routine nursing or cluster nursing. They will be followed up for 3 months after 4 weeks of treatment. Observation indicators include: The total effective rate of cardiac function improvement, Minnesota Living with Heart Failure Questionnaire, left ventricular ejection fraction, N-terminal pro-brain natriuretic peptide, 6-minute walk test, adverse reaction, etc. Data were analyzed using the statistical software package SPSS version 25.0.

**Discussion:**

This study will evaluate the effects of cluster nursing on cardiac function and quality of life of CHD patients with chronic heart failure. The results of this study will provide clinical basis for establishing reasonable and effective nursing programs for CHD patients with chronic heart failure.

## 1 Introduction

With the changes in people's diet structure and life schedule, the incidence of cardiovascular diseases is increasing and it has become the deadliest disease in the world.^[1,2]^ Chronic heart failure is the terminal stage of the development of many cardiovascular diseases.^[3]^ According to the American epidemiological survey, there are currently 6 million patients with chronic heart failure in the United States, and this number is expected to increase to 9 million by 2030.^[4]^ At present, there has been no breakthrough in the treatment of coronary heart disease (CHD) complicated with chronic heart failure. Due to the particularity of the disease, the quality of life of patients is seriously reduced, and life is even endangered, causing huge burden to patients and their families.^[5]^ Therefore, how to improve the quality of life of patients, improve the state of cardiac function, and reduce the case fatality rate are the directions that clinical workers need to focus on.

The treatment of heart disease complicated with chronic heart failure cannot only rely on drugs, high-quality nursing work also plays an important role in the treatment.^[6]^ Studies have shown that patients with CHD complicated with heart failure can enhance their therapeutic effect and improve their quality of life on the basis of routine medication combined with quality nursing.^[7]^

Cluster nursing is an intensive care unit idea jointly proposed by the American Institute of Health and the Association of Volunteer Hospitals. It is a nursing program that integrates a series of evidence-based nursing interventions.^[8]^ Cluster nursing consists of 3 to 5 nursing measures with strong operability, simplicity, and easy acceptance in clinical practice, each of which has been verified by clinical practice and can effectively improve the symptoms of patients. Clinical observation shows that the synergistic effect of these interventions is better than that of a single measure.^[9]^ At present, cluster nursing has been applied to patients after surgery and intensive care unit, and has received positive feedback.^[10,11]^ However, there is still a lack of high-quality clinical studies to explore the impact of cluster nursing on patients with coronary heart failure. Therefore, this study will explore the effects of cluster nursing on cardiac function and quality of life in CHD patients with chronic heart failure.

## 2 Materials and methods

### 2.1 Study design

**Figure 1:**
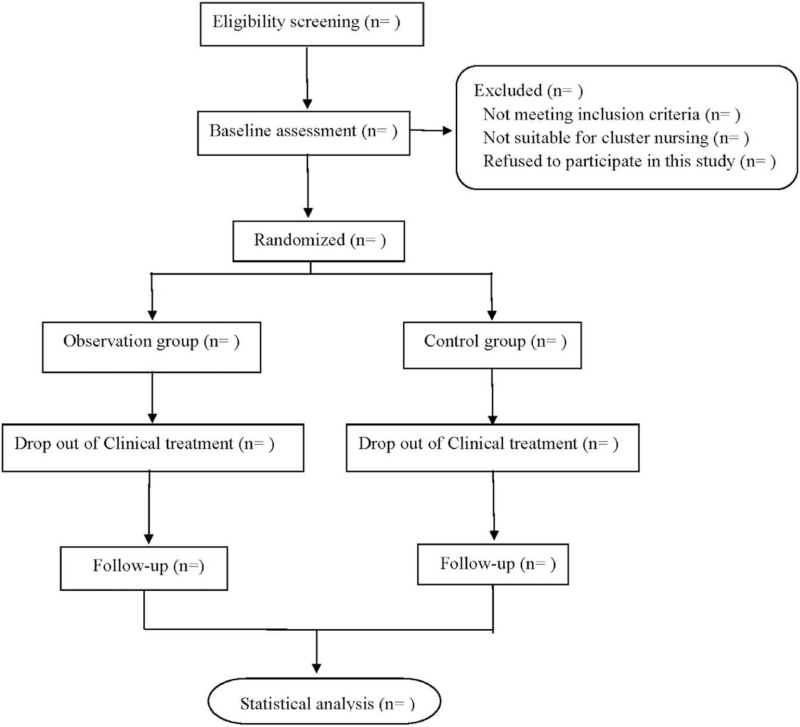
Flow diagram.

This is a prospective randomized controlled trial to study the effects of cluster nursing on cardiac function and quality of life of CHD patients with chronic heart failure. This study will follow Consolidated Standards of Reporting Trials,^[12]^ and the flow chart is shown in Figure 1.

### 2.2 Ethics and registration

This research scheme is in line with the Helsinki Declaration and approved by the Clinical Research Ethics Committee of our hospital. This experiment is registered in the open science framework (registration number: DOI 10.17605/OSF.IO/WQMNJ). Before being randomly divided into groups, all patients need to sign a written informed consent form, and they are free to choose whether or not to continue the trial at any time.

### 2.3 Sample size

The calculation of sample size is based on mean and standard deviation of scores of Minnesota Living with Heart Failure Questionnaire^[13]^ after treatment. According to the results of the pilot study, the observation group will be 27.91 ± 4.31, and the control group will be 31.12 ± 4.85. Set α = 0.025, unilateral test, β = 0.20. According to the calculation of PASS15.0 software, 34 participants are needed in each group, the estimated withdrawal rate is 10%, so that 38 participants will be included in each group.

### 2.4 Patients

Inclusion criteria: ① Patients with occlusive myocardial infarction who meet the diagnostic criteria for CHD, which the stenosis rate of at least one of the main coronary arteries is more than 50%; ② Patients who are in line with the diagnostic criteria of chronic heart failure,^[14,15]^ and Doppler ultrasound suggests that left ventricular ejection fraction <50%. ③ Age 40 to 79 years old; ④ Patients with clear awareness and good compliance; ⑤ Patients who have signed the informed consent form.

Exclusion criteria: ① Patients with other diseases such as congenital heart disease that cause heart failure; ② Patients complicated with other serious diseases such as malignant tumors; ③ Patients with the presence of other heart lesions such as severe arrhythmia; ④ Patients with severe mental illness.

### 2.5 Study design

Subjects meeting inclusion and exclusion criteria will be randomly assigned to control and observation groups in a 1:1 ratio using a central network randomization tool. Random sequences will be generated using SAS 9.3 software (SAS Institute, Cary, NC) by independent statisticians not involved in the treatment process and statistical analysis of the data. Randomization will be performed without any stratification. The clinical study coordinator enters the subject information on a tablet computer and is given a random number. The research assistant gets the subject assignment information from the computer. Throughout the trial, the research assistant is responsible for recruiting, screening, and assigning random numbers to those who have been enrolled in the study. None of the research assistants, intervention supervisors, or those performing statistical analysis are aware of the group assignment.

### 2.6 Intervention measures

Both groups will receive the same drug regimen and different care regimens during treatment.

Observation group: receive cluster nursing, including: ① expectoration nursing: Introduce the importance of expectoration to patients, improve their compliance, and correctly guide the cough method. If effective cough expectoration cannot be achieved, expectoration can be carried out by slapping the back, beating and aerosol inhalation.^[16]^② Walking training: Nursing staff instructs patients to walk a short distance indoors, and the time and distance of walking can be extended after patients are gradually adapted to it. During the training, patients will stop training immediately if they have dyspnea or chest pain.^[17]^③ Posture nursing: In order to ensure normal and smooth breathing of patients, patients’ posture should be dynamically adjusted in combination with their physical status, mainly sitting and semi-reclining.^[18]^④ Psychological nursing: provide psychological counseling and care to patients, eliminate their negative emotions, and encourage them to maintain their optimistic attitude.^[19]^⑤ Dietary guidance: Patients will be given dietary guidance to reduce the intake of stimulating food, eat less and eat more frequently, and maintain a healthy and reasonable diet.^[20]^

The control group will receive routine nursing mode, including daily nursing and health guidance.

### 2.7 Evaluation criteria and efficacy judgment

(1) Main outcome indicators: ① Total rate of improvement in cardiac function (refer to the principle of clinical research on treating heart failure with new Chinese Medicine)^[21]^; Excellent: heart failure is essentially ameliorated or the NYHA classification increased by at least 2 levels; Valid: NYHA classification increased by 1 level; Invalid: NYHA classification remain the same before and after the treatment; Worsened: NYHA classification decrease by at least. ② Minnesota Living with Heart Failure Questionnaire,^[13]^ the higher the score, the worse the patient's quality of life.
(2) Secondary outcome indicators: left ventricular ejection fraction, N-terminal pro-brain natriuretic peptide, tumor necrosis factor-α, IL- 6, 6-minute walk test.
(3) Adverse reactions: including abnormal liver and kidney function and any uncomfortable symptoms (such as dizziness, nausea, etc) during treatment.

The above observation indicators will be collected on the day before and after treatment. All patients will be followed up for 3 months, and data will be collected according to the same criteria in the first and third months.

### 2.8 Data collection and management

The data involved in this study will be collected by 2 research assistants, and summarize and analyze by a statistician and a data analyst. Personal information about potential and registered participants will be collected, shared, and kept in a separate storage room to protect confidentiality before and after the trial.

### 2.9 Statistical analysis

SPSS 25.0 (Chicago, IL) software will be used for statistical analysis of the collected data. Chi-square test will be used for counting data; measurement data is represented by $\bar{\text{x}}\pm \text{S}$, the independent sample *t* test is used for normal distribution, non-parametric test is used for skewness distribution. When *P* < .05, the difference is statistically significant.

## 3 Discussion

CHD chronic heart failure is characterized by prolonged disease and poor prognosis, which has a serious impact on the quality of life of patients and even endangers their lives. High-quality nursing is the basis to ensure the therapeutic effect.^[22,23]^

Cluster nursing standardizes various nursing processes based on evidence-based evidence, which can reduce the causes of heart failure, reduce the cardiac load of patients, improve the condition of patients, restore the quality of life of patients, and improve the prognosis of patients.^[24,25]^ However, due to few clinical studies on related topics, cluster nursing has not been widely used in clinical nursing of CHD chronic heart failure. Therefore, this study will explore the impact of cluster nursing on the cardiac function and quality of life of CHD patients with chronic heart failure. At the same time, this study has the following limitations: as this is a single-center randomized controlled study, the included population is regionalized, and the results may be biased to some extent; due to the factors of the intervention program, this study could not achieve strict double blindness, which may have some influence on the results.

## Author contributions

**Data curation:** Qian Jin, Yi Zhou.

**Formal analysis:** Qian Jin, Delu Yin.

**Funding acquisition:** Yiling Wu.

**Investigation:** Qian Jin, Hong He.

**Resources:** Yi Zhou, Delu Yin.

**Supervision:** Yiling Wu, Delu Yin.

**Writing – review & editing:** Yonghua Liu, Yiling Wu.

**Writing - review & editing:** Yonghua Liu, Yiling Wu.
